# En Balance: The Contribution of Physical Activity to the Efficacy of Spanish Diabetes Education of Hispanic Americans with Type 2 Diabetes

**DOI:** 10.1155/2020/4826704

**Published:** 2020-04-19

**Authors:** Dequina A. Nicholas, Lorena M. Salto, Kristen Lavelle, Joy Wilson, W. Lawrence Beeson, Anthony Firek, William H. R. Langridge, Zaida Cordero-MacIntyre, Marino De Leon

**Affiliations:** ^1^Center for Health Disparities and Molecular Medicine, Loma Linda University School of Medicine, 11085 Campus Street, Loma Linda, CA 92350, USA; ^2^University of California San Diego, School of Medicine, Department of Obstetrics, Gynecology, and Reproductive Sciences, 9500 Gilman Drive, La Jolla, CA 92093, USA; ^3^Division of Biochemistry, Department of Basic Sciences, Loma Linda University School Medicine, 11085 Campus Street, Loma Linda, CA 92350, USA; ^4^California Pacific Medical Center, 2333 Buchanan Street, San Francisco, CA 94115, USA; ^5^Department of Epidemiology and Biostatistics, Loma Linda University School of Public Health, 24951 North Circle Drive, Loma Linda, CA 92350, USA; ^6^Section of Endocrinology, JL Pettis Memorial VA Medical Center, 11201 Benton St, Loma Linda, CA 92357, USA; ^7^Center for Nutrition, Healthy Lifestyle and Disease Prevention, School of Public Health, Loma Linda University, 24951 North Circle Drive, Loma Linda, CA 92350, USA; ^8^Whittier College, 13406 E Philadelphia Street, Whittier, CA 90602, USA; ^9^Division of Physiology and Pharmacology, Department of Basic Sciences, Loma Linda University School Medicine, California, 11175 Campus Street, Loma Linda, CA 92350, USA

## Abstract

**Purpose:**

*En Balance*, a culturally sensitive diabetes education program, improves glycemic control in Hispanics with type 2 diabetes. The program emphasized diet, physical activity, and other factors important for glycemic control. However, the individual contributions of these education factors are unclear. The purpose of this study is to assess the contribution of physical activity to the success of *En Balance* in improving the health of Mexican Americans with type 2 diabetes.

**Methods:**

A retrospective study was conducted with plasma samples collected pre- and post-3-month study. Samples from 58 (18 males and 40 females) Hispanic subjects with type 2 diabetes were analyzed for the concentration of kynurenines, known to decrease in response to exercise. After three months, health outcomes for the active group (decreased kynurenines) and the rest of the cohort were evaluated by paired Wilcoxon signed-rank test.

**Results:**

Half of the subjects had increased kynurenine levels at the end of the educational program. We found that the subjects in the active group with decreased kynurenine concentrations displayed statistically greater improvements in fasting blood glucose, A1C, cholesterol, and triglycerides despite weight loss being higher in the group with increased kynurenine concentrations.

**Conclusions:**

*En Balance* participants with decreased kynurenine levels had significantly improved glycemic control. These data suggest that physical activity significantly contributes to the success of the *En Balance* education program. This analysis indicates that diabetes public health educators should emphasize the benefit of physical activity on glycemic control even in the absence of major weight loss.

## 1. Introduction

Type 2 diabetes (T2DM) is one of the fastest growing public health problems facing the United States today. The grim statistics associated with T2DM make a strong case for devoting resources to prevention, interventions, and finding a cure. According to the most recent comprehensive assessment of the impact of diabetes in the U.S., the prevalence of T2DM in the U.S. is 30.3 million Americans, or 9.4% of the population, and rising [1]. In 2015 alone, there were 1.5 million new diagnoses. If current trends continue, 1 in 3 Americans will likely have diabetes by 2050 [2]. Diabetes can lead to serious comorbid conditions and complications, including heart disease, kidney failure, and blindness, making it the seventh leading cause of death in the U.S. and resulting in a substantial financial burden on society [3]. The total cost of the diagnosed T2DM in 2012 in the U.S. was $245 million, and the average medical expenditures among people with diagnosed diabetes were 2.3 times higher than would be predicted in the absence of diabetes [1].

T2DM or non-insulin-dependent or adult-onset diabetes accounts for 90 to 95% of the total diagnosed cases of diabetes in adults. While T2DM affects millions of people across the country, it does so disproportionately with respect to ethnicity. The rates of the diagnosed T2DM by ethnicity are 7.6% of non-Hispanic whites, 9.0% of Asian Americans, 12.8% of Hispanics, 13.2% of non-Hispanic blacks, and 15.9% of American Indians/Alaskan Natives [3].

The percentage of Hispanics diagnosed with T2DM (12.8%) is considerably higher than the national average (9.3%) and is particularly concentrated in the state of California, one of the regions with the highest number of Hispanics in the country. Among the U.S. states, California has the greatest number of new cases of diabetes annually [4]. Compared with non-Hispanic Whites, Hispanics living in California have twice the prevalence of T2DM and are twice as likely to die from the disease.

Hispanics are more likely than non-Hispanic whites to have a cluster of risk factors for developing T2DM referred to collectively as metabolic syndrome [5]. Risk factors involved in this syndrome are cardiometabolic abnormalities, and examples include hypertension, abdominal obesity, and dyslipidemia. Additionally, Hispanics face a variety of social and economic barriers to receiving healthcare, which lead to their overall compromised health status. Compared with non-Hispanic whites, Hispanics have a lower average income as well as lower levels of education and literacy [6].

To address this disparity, diabetes education programs have formed, including our program *En Balance*, a culturally and language-sensitive Hispanic diabetes education program in San Bernardino County. After three months in the program, participants had significantly improved glycemic control [7, 8], lipid profiles [8, 9], and decreased obesity [7]. Of note, the program, though it did not prescribe any specific exercise regimen, also resulted in an increase in moderate and high intensity physical activity as determined by the validated Arizona Activity Frequency Questionnaire [10]. Hispanic Americans report some of the lowest levels of physical activity in the United States [11, 12]. Therefore, it is important to understand the impact of increased physical activity on glycemic control in this population. However, due to common self-reporting biases towards more exercise, the relationship between physical activity and the efficacy of the *En Balance* program remains unclear [13, 14]. Kynurenine, a degradation product of the essential amino acid tryptophan, is known to be elevated in T2DM and can be decreased by exercise [15–17]. Using post hoc analysis, this study is aimed at using changes in kynurenine levels as an indicator of physical activity to determine its contribution to the success of the *En Balance* program.

## 2. Materials and Methods

### 2.1. Research Design and Methods

This study consisted of analysis of plasma samples from a 3-month diabetes education intervention (*En Balance*) designed to promote improved T2DM management in Hispanic adults [7–9, 18]. We measured kynurenines in these samples to determine which subjects had decreased kynurenine after 3 months, and then reassessed the original primary outcomes of the *En Balance* study. These outcomes included fasting blood glucose, A1C, and body composition. A total of 73 Hispanic males and females with T2DM met the *En Balance* participation criteria as previously described [8, 9]. Of these 73 participants, a subgroup of 58 participants with available plasma was analyzed.

### 2.2. Ethics and Informed Consent (*En Balance Study*)

The Loma Linda University Institutional Review Board (IRB) approved the *En Balance* study protocol, and all participants gave written informed consent to participate. Signed consent forms for the study are stored in locked filing cabinets and cannot be linked to participant data according to Loma Linda University IRB protocol.

### 2.3. Evaluative Measures (*En Balance Study*)

#### 2.3.1. Glucose, A1C, and Insulin

Two blood samples (12-14 hr fasting) were drawn from the participants at both baseline and 3 months and analyzed for glucose, A1C, and insulin. Additional samples were stored frozen at -80°C for future analysis.

#### 2.3.2. Anthropometric Measures and Body Fat Composition

Anthropometric measures (height, weight, waist circumference, hip circumference, and waist/hip ratio) were assessed at baseline and 3 months as previously described [18, 19]. Body composition was assessed at baseline and at 3 months using a TANITA scale (Detecto, Web City, Missouri), bioelectric impendence technology, and a fan beam dual X-ray absorptiometry (DXA) Hologic Discovery A software version 12.6 (Waltham, MA) as previously described [8, 9].

### 2.4. Plasma Samples for Detection of Inflammatory Markers

Plasma samples from 58 *En Balance* participants were available for L-kynurenine testing. The baseline characteristics of these participants are presented in Table 1 with the exception of missing participant data. All participants had paired plasma samples from baseline and 3 months available for longitudinal analysis.

### 2.5. Kynurenine Assay

To measure kynurenine in plasma samples, the standard kynurenine assay mixture (100 *μ*l total volume) contained 50 mM potassium phosphate buffer (pH 6.5), 20 mM ascorbate, 10 *μ*M methylene blue, and 100 *μ*g/ml catalase plus 50 *μ*l serum. The reaction was initiated by adding the indoleamine-2,3-dioxygenase substrate L-tryptophan (400 *μ*M) and terminated after 1-hour incubation at 37°C. A volume of 10 *μ*l of 30% trichloroacetic acid was added to the mixture followed by incubation of the mixture at 50°C for 30 minutes to terminate the reaction and to hydrolyze the N-formyl kynurenine cleaved by IDO1 to L-kynurenine. The optical density of the samples was measured at 490 nm. A standard curve of defined kynurenine concentrations (0–100 *μ*M) was plotted against the absorbance values. Concentration for each sample (in duplicate) was determined from the standard curve as previously described [20, 21].

### 2.6. Statistical Analysis

Statistical analyses were calculated using SPSS for Windows version 22 (SPSS, Inc., Chicago, Illinois) with type I error set at *α* = 0.05. The data are presented as mean ± SD. Univariate and bivariate analyses are reported at baseline and at 3 months. The Wilcoxon signed-rank test was used to identify baseline to 3-month paired differences. Mann–Whitney test was used for nonparametric unpaired data analysis of changes in kynurenine levels. GraphPad Prism 7 was used to generate figures.

## 3. Results

Table 1 presents the baseline characteristics of *En Balance* participants (*n* = 58). Study participants included 40 women and 18 men, with most participants being age ≥ 40 years and BMI ≥ 25. Nearly 70% of the participants were female. Of the baseline characteristics, only hip circumference was significantly different between males and females.

Table 2 summarizes the baseline, 3-month, and 3-month mean changes in anthropometric measures, body composition, and clinical evaluators of T2DM. After 3 months of education, a significant reduction in means was observed for fasting blood glucose (-21.0, *P* = 0.001), A1C (−0.90%, *P* < 0.001), total cholesterol (−8.7 mg/dl, *P* = 0.049), cholesterol : HDL ratio (−0.32 mg/dl, *P* = 0.002), waist circumference (-2.4 cm, *P* = 0.001), and DXA : trunk fat (−0.4 kg, *P* = 0.001) as compared with baseline. There was no significant mean change in insulin, triglycerides, or BMI.

**Figure 1:**
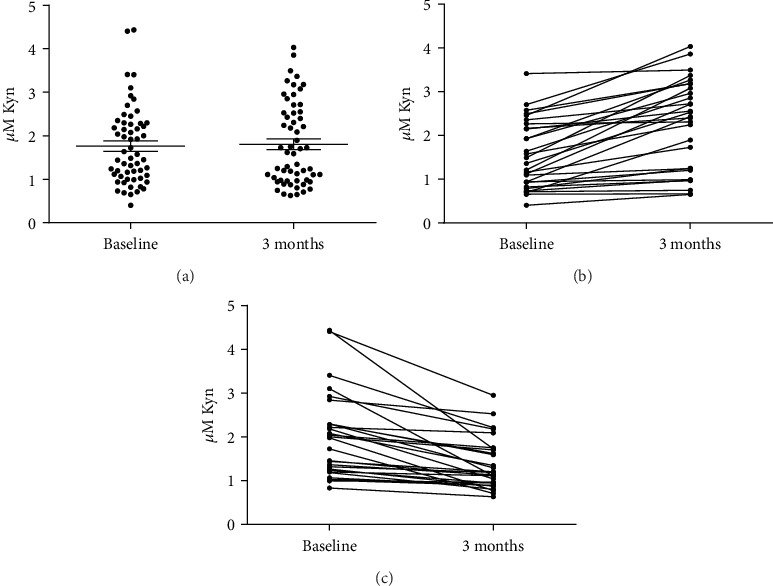
Kynurenine levels in plasma of Hispanic *En Balance* participants with T2DM following 3 months of diabetes education. (a) Kynurenine levels for all study participants (*n* = 58) are plotted (mean ± SEM). (b) Paired data from the 29 *En Balance* participants whose plasma kynurenines decreased after 3 months. (c) Paired data from the 29 *En Balance* participants whose plasma kynurenines increased after 3 months. The black lines connect data points belonging to the same participant.

To divide the *En Balance* data into “active” and “test” groups, we assayed kynurenines in plasma samples from baseline and 3 months. Several publications have demonstrated that exercise reduces circulating kynurenine by increasing the metabolism of kynurenine to kynurenic acid via an enzyme in the skeletal muscle called kynurenine aminotransferases [15–17]. In particular, persons with T2DM have significantly lowered plasma kynurenine after recovering from 30 min of continuous exercise [15]. In addition, several exercise programs including resistance training and multiple modes of intensity over periods of weeks resulted in decreased serum or plasma KYN in diseased subjects [15, 22, 23]. This is likely due to the KYN metabolic pathway being over activated in several disease states including type 2 diabetes [22, 24, 25].  Figure 1(a) depicts the kynurenine concentration in plasma from *En Balance* participants at baseline and 3 months after the intervention. Overall, there was no change. Half of the participants exhibited a decrease in kynurenine after 3 months (Figure 1(b)), while the other half showed an increase in kynurenine (Figure 1(c)). Because exercise decreases kynurenine, we separated the participant data into two groups: active group (decreased kynurenine) or test group (increased kynurenine).

Table 3 summarizes the baseline, 3-month, and 3-month mean changes in anthropometric measures, body composition, and clinical evaluators of T2DM in the active group (decreased kynurenine, Figure 1(b)) and the test group (increased kynurenine, Figure 1(c)). Participants who were physically active showed a statistically significant reduction in triglycerides (-33.5 mg/dl, *P* = 0.014) and LDL cholesterol (-11.3 mg/dl, *P* = 0.032) which was not apparent for the whole group (Table 2). The active, but not the test group, had reductions in fasting blood glucose and total cholesterol. The test group had decreased DXA : trunk fat and BMI while both groups had significantly improved A1C.

## 4. Discussion

This study uses changes in plasma kynurenines as a proxy to determine the contribution of physical activity to the efficacy of the *En Balance* education program. After 3 months of diabetes education in a Hispanic T2DM cohort, changes in kynurenine levels were used to identify those participants who were physically active. Using this approach, we were able to determine indicators of glycemic control and metabolic health that are most impacted by physical activity.

Using reduced KYN levels as a proxy for physical activity requires consideration of several factors because KYN reduction in response to exercise is an indirect response. The regulation of KYN levels is modulated by the expression of two enzymes, indoleamine-2,3-dioxygenase (IDO) and tryptophan-2,3-dioxygenase (TDO or TDO2), which regulate the first and rate-limiting step of the KYN pathway. Increases in IDO and TDO result in increased KYN while the converse is true. IDO is expressed by several cell types throughout the body such as stromal, vascular, and immune cells, especially antigen-presenting cells, and its expression is induced by some inflammatory cytokines including IL-6, TNF-*α*, and IFN gamma, with IFN gamma being the strongest inducer [26, 27]. TDO is expressed in the liver and is induced by cortisol [28, 29]. Therefore, lowered KYN levels are caused by a reduction in inflammation or a reduction in stress responses (cortisol). Increased physical activity over time, independent of weight loss, reduces chronic inflammation [30] associated with T2DM [31] and is also known to reduce cortisol. There are some reports that extreme calorie restriction can reduce plasma KYN [32, 33], due to the low availability of tryptophan. In our study, participants did not significantly reduce their overall caloric intake over the 3-month period as assessed by a food frequency questionnaire [8]. Therefore, we can attribute changes in plasma KYN to physical activity and the presumed impact of physical activity on chronic inflammation.

The precise role of kynurenines in the pathogenesis of T2DM is unclear. Current literature suggests that increased levels of kynurenines associated with T2DM are pathogenic [24, 34, 35]. In their 1985 review, Connick and Stone proposed one of the first hypotheses concerning the role of kynurenines in diabetes, postulating that kynurenines may possibly be involved in causation of diabetes mellitus [35]. This observation has been corroborated by recent findings. In his first publication on this topic, Oxenkrug proposed a “kynurenine hypothesis of insulin resistance and its progression to T2DM” [34]. Our data agrees with this hypothesis because glycemic control is improved to a greater extent in those participants that had reduced plasma kynurenine levels after 3 months in the *En Balance* program. However, our data do not preclude the probable explanation that inflammation may be upstream of dysregulated kynurenines in T2DM. Chronic inflammation in T2DM is well established [36]. In this study, makers of inflammation were not measured, which is therefore a limitation to interpreting the mechanism linking physical activity to changes in plasma kynurenines.

A most intriguing observation is that the reduced kynurenine group had improved lipid profiles while the higher kynurenine test group and the overall cohort did not (Tables 2 and 3). This result underscores the importance of physical activity in regulating lipid metabolism and validates the approach of using changes in plasma kynurenine levels to assess activity status. Reports on the direct effect of exercise on blood lipid levels commonly show a decrease in total cholesterol, LDL cholesterol, and the ratio of cholesterol : HDL [37–39]. Similar changes in blood lipids were found in the decreased kynurenine group but not in the increased kynurenine group.

While the reduced kynurenine group did not lose as much weight as the test group did, their metabolic status was improved to a greater extent (Table 3). These experimental results are important for diabetes educators to consider. Due to the slow process of weight loss, education programs that emphasize the goal to be weight loss instead of healthy may discourage those participants who do not experience changes in weight. Despite moderate decreases in trunk fat and BMI, *En Balance* participants had significant results in glycemic control in just a 3-month period. The data presented here demonstrate that physical activity is a major contributing factor to these beneficial outcomes.

While this study is limited by its retrospective nature and relatively small sample size, it raises important points to consider about diabetes education and methods for tracking physical activity. In the cohort from this study, Wheeler et al. found little differences in glycemic control between *En Balance* participants who increased energy expenditure more than 300 kcal or less than 300 kcal [10]. This result is probably due to errors in self-reporting. Over reporting for physical activity is a common occurrence in epidemiological studies for a variety of reasons including external pressures like social approval [10, 11]. Changes in kynurenine levels offer a viable alternative or compliment to self-reporting for epidemiological studies analyzing exercise habits. Further validation of changes in kynurenine levels as a proxy for exercise or physical activity is warranted to complement studies aimed at understanding the relationship of kynurenines to T2DM and as indicators of glycemic control.

## 5. Conclusions

In conclusion, this study demonstrates that increased physical activity generated by the *En Balance* program is an important factor for participant achievement of a better health outcome. Those participants who increased physical activity throughout the duration of the program, as measured by a decrease in kynurenine levels, demonstrated improved lipid profiles and better management of blood glucose despite comparatively minimal weight loss. Based on the experimental results generated by this study, it is important for diabetes educators to emphasize to their patients the benefits of physical activity for improved glycemic control, especially for the Hispanic American community who historically report inadequate levels of physical activity.

## Figures and Tables

**Table 1:** Participant characteristics at baseline (*n* = 58).

	Males (*n* = 18) % (*n*)	Females (*n* = 40)^a^ % (*n*)
Age (years)		
30-39	11.1% (2)	10.0% (4)
40-49	33.3% (6)	25.0% (10)
50-59	27.7% (5)	45.0% (18)
60-69	27.7% (5)	12.5% (5)
70-79	—	7.5% (3)
Mean ± SD	52.1 ± 9.7	52.9 ± 10.2
BMI (kg/m^2^)		
18.5-24.9	11.1% (2)	15.4% (6)
25.0-29.9	55.5% (10)	30.8% (12)
30.0 and higher	33.3% (6)	53.8% (21)
Mean ± SD	29.7 ± 5.2	32.8 ± 7.4
Waist circumference (cm)		
70-79	—	7.5% (3)
80-89	11.1% (2)	12.5% (5)
90-99	38.8% (7)	32.5% (13)
100-109	38.8% (7)	20.0% (8)
110-119	—	20.0% (8)
120-129	5.6% (1)	7.5% (3)
130-139	5.6% (1)	—
Mean ± SD	101.8 ± 11.8	100.2 ± 13.2
Hip circumference (cm)		
80-89	5.6% (1)	2.5% (1)
90-99	22.2% (4)	25.0% (10)
100-109	55.5% (10)	32.5% (13)
110-119	11.1% (2)	10.0% (4)
120-129	—	12.5% (5)
130-139	5.6% (1)	12.5% (5)
140-149	—	5.0% (2)
Mean ± SD	102.8 ± 9.9^∗^	111.5 ± 15.5^∗^

BMI = body mass index. Percentages may not add to one hundred due to rounding. ^a^*n* = 39 for female BMI data. ^∗^Statistically significant mean difference between males and females (*P* = 0.013).

**Table 2:** Participant blood glucose, lipid, and body composition profiles at baseline and at three months (*n* = 58).

	Baseline	Three months	Mean difference	*P* value
	Mean ± SD	Mean ± SD	± SD	
Fasting blood glucose (mg/dl)	166.8 ± 72.2	145.7 ± 58.6	-21.0 ± 45.0	0.001^∗^
A1C (%)	8.1 ± 2.3	7.2 ± 1.6	-0.9 ± 1.4	<0.001^∗^
Insulin (*μ*U/ml)	12.0 ± 8.5	13.1 ± 10.8	-1.0 ± 6.4	0.200
HOMA-IR^a^	4.52 ± 2.83	4.39 ± 3.06	-0.12 ± 2.24	0.673
Total cholesterol (mg/dl)	194.9 ± 40.3	186.2 ± 47.2	8.7 ± 33.2	0.049^∗^
HDL cholesterol (mg/dl)	46.2 ± 9.2	48.0 ± 10.7	-1.8 ± 7.2	0.061
LDL cholesterol (mg/dl)	125.5 ± 35.7	118.6 ± 37.3	-6.8 ± 26.9	0.059
Cholesterol : HDL ratio	4.3 ± 1.0	3.9 ± 1.0	-0.32 ± 0.77	0.002^∗^
Triglycerides (mg/dl)	199.1 ± 115.0	183.1 ± 115.6	-16.0 ± 78.1	0.123
Waist circumference (cm)	100.6 ± 12.7	98.2 ± 12.1	-2.4 ± 5.1	0.001^∗^
Hip circumference (cm)	108.7 ± 14.4	108.3 ± 13.2	-0.4 ± 3.9	0.364
BMI (kg/m^2^)	31.7 ± 6.9	31.4 ± 6.6	-0.3 ± 1.4	0.055
DXA : trunk fat (kg)	15.7 ± 6.1	15.3 ± 5.9	-0.4 ± 1.0	0.001^∗^

BIA = bioelectrical impedance analysis; BMI = body mass index; DXA = dual-energy X-ray absorptiometry. ^a^Homeostasis Model Assessment of Insulin Resistance (HOMA − IR) = [fasting insulin (mU/l) × fasting glucose (mmol/l)]/22.5. ^∗^*P* < 0.05 and based on paired sample Mann–Whitney test.

**Table 3:** Participant blood glucose, lipid, and body composition profiles at baseline and at three months (*n* = 58).

	Baseline	Three months	Mean difference	*P* value
	Mean ± SD	Mean ± SD	± SD	
Fasting blood glucose (mg/dl)				
↓KYN	164.96 ± 79.8	135.2 ± 59.7	-29.7 ± 41.7	<0.001^∗^
↑KYN	170.1 ± 64.8	155.25 ± 56.2	-14.8 ± 45.5	0.101
A1C (%)				
↓KYN	8.3 ± 2.6	7.2 ± 1.6	-1.06 ± 1.7	0.002^∗^
↑KYN	8.1 ± 2.1	7.4 ± 1.8	-0.8 ± 1.1	0.001^∗^
Insulin (*μ*U/ml)				
↓KYN	13.4 ± 10.0	14.7 ± 12.8	1.4 ± 6.6	0.265
↑KYN	11.0 ± 6.5	11.3 ± 8.0	0.3 ± 5.7	0.784
HOMA-IR^a^				
↓KYN	4.7 ± 2.6	4.4 ± 3.0	-0.26 ± 1.8	0.437
↑KYN	4.5 ± 3.0	4.21 ± 3.1	-0.25 ± 2.3	0.564
Total cholesterol (mg/dl)				
↓KYN	195.1 ± 38.0	179.2 ± 44.5	-15.9 ± 32.0	0.012^∗^
↑KYN	193.8 ± 42.7	194.9 ± 48.7	-1.17 ± 29.8	0.838
HDL cholesterol (mg/dl)				
↓KYN	46.3 ± 9.4	47.5 ± 9.9	1.17 ± 5.8	0.287
↑KYN	45.67 ± 8.8	48.3 ± 11.6	2.6 ± 8.5	0.119
LDL cholesterol (mg/dl)				
↓KYN	124.7 ± 35.3	113.5 ± 38.6	-11.3 ± 26.9	0.032^∗^
↑KYN	125.3 ± 36.3	125.7 ± 34.6	0.5 ± 21.8	0.913
Cholesterol : HDL ratio				
↓KYN	4.3 ± 1.0	3.9 ± 1.0	-0.5 ± 0.8	0.003^∗^
↑KYN	4.3 ± 1.0	4.2 ± 1.1	-0.2 ± 0.7	0.275
Triglycerides (mg/dl)				
↓KYN	195.0 ± 88.7	161.4 ± 61.9	-33.5 ± 69.3	0.014^∗^
↑KYN	208.7 ± 135.1	208.1 ± 148.6	-0.6 ± 82.9	0.970
BMI (kg/m^2^)				
↓KYN	31.8 ± 7.3	31.7 ± 7.0	-0.1 ± 1.9	0.808
↑KYN	31.8 ± 6.5	31.2 ± 6.2	-0.7 ± 0.6	<0.001^∗^
DXA : trunk fat (kg)				
↓KYN	16.1 ± 6.6	15.8 ± 6.5	-0.3 ± 1.1	0.207
↑KYN	15.2 ± 5.5	14.5 ± 5.5	-0.7 ± 0.9	<0.001^∗^

BIA = bioelectrical impedance analysis; BMI = body mass index; DXA = dual-energy X-ray absorptiometry. ^a^Homeostasis Model Assessment of Insulin Resistance (HOMA − IR) = [fasting insulin (mU/l) × fasting glucose (mmol/l)]/22.5. ^∗^*P* < 0.05 3 months to baseline, paired sample Mann–Whitney test. ↓KYN = subjects whose plasma kynurenines decreased after the 3-month intervention. ↑KYN = subjects whose plasma kynurenines increased after the 3-month intervention.

## Data Availability

The guarantors of this work are Z.C., W.R.L., and M.D. and, as such, have full access to all the data in the study and take responsibility for the integrity of the data and the accuracy of the data analysis. Data supporting the results herein are available upon request to the corresponding author.
